# Association between Cardiorespiratory Fitness and Metabolic Syndrome in Korean Older Adults

**DOI:** 10.3390/ijerph19063671

**Published:** 2022-03-19

**Authors:** Shinuk Kim

**Affiliations:** Department of Smart Information Communication Engineering, Sangmyung University, Cheonan 330-720, Korea; kshinuk@gmail.com; Tel.: +82-041-550-5452

**Keywords:** cardiorespiratory fitness, metabolic disorders, older men, risk stratification

## Abstract

Background: Little is known about the relationship between non-exercise-based estimation of cardiorespiratory fitness (eCRF) and metabolic syndrome (MetS) in Korea. The current study examined the prognostic role of eCRF in the risk stratification of MetS in a representative sample of Korean older adults (1822 men and 3069 women). Methods: The data used in the current study were extracted from the Korea National Health and Nutrition Examination Surveys IV and V. eCRF was obtained using a previously validated algorithm. MetS was defined according to the National Cholesterol Education Program definition with the acceptance of a Korean-specific waist circumference cutoff point. Results: Lower eCRF was significantly correlated with abnormalities in several components of MetS, including abdominal obesity, elevated glucose, elevated triglycerides, and decreased high-density lipoprotein cholesterol. Furthermore, there was an inverse linear relationship between MetS prevalence and eCRF levels; higher eCRF was significantly and independently associated with lower prevalence of MetS. Conclusion: The current findings suggest that eCRF can be adopted as a prognostic measure in determining the risk for MetS for elderly persons.

## 1. Introduction

Metabolic syndrome (MetS) represents the preclinical feature of clustered metabolic risk factors that contributes to an increased risk for type 2 diabetes (T2D) and/or cardiovascular disease (CVD) in both developed and developing countries [1,2]. Components of MetS include central obesity, elevated blood pressure, elevating fasting glucose, elevated triglycerides, and decreased high-density lipoprotein cholesterol [3]. Etiologically, excessive caloric intake and physical inactivity have been considered as two major modifiable risk factors for the global epidemic of MetS [4]. The relationships between obesity and physical inactivity and/or poor physical fitness with MetS have been observed in Korean adults [5,6,7].

Cardiorespiratory fitness (CRF) reflects the respiratory and circulatory capacity of delivering oxygen into skeletal muscles for energy production during sustained physical activity. The prognostic role of CRF for the prediction of MetS has been substantially and consistently reported in the literature [8,9]. However, the needs for an ergometer, a metabolic cart, trained personnel, sufficient time, and others limit the objective measurement of CRF, especially among elderly persons.

Alternatively, CRF can be estimated using regularly checked health parameters with an acceptable accuracy [10,11], and non-exercise-based estimation of CRF (eCRF) has a prognostic value for the prediction of morbidity and mortality in different populations [12,13]. Furthermore, eCRF is inversely associated with the risk of non-alcoholic fatty liver disease (NAFLD) in Korean adults [14].

One-third of Korean adults suffer from MetS, and the prevalence of MetS is on the rise with advanced age [15]. In South Korea, population aging is growing at the fastest pace among the Organization for Economic Co-operation and Development (OECD) countries (https://data.oecd.org/pop/elderly-population.htm (accessed on 7 September 2021)), implying a substantial health burden and medical cost to Korean society. Therefore, considering its steadily rising prevalence in the face of a rapidly aging society, MetS will continue to represent a key public health issue in South Korea, especially among elderly Koreans.

To the best of our knowledge, only a handful of studies have reported an association between eCRF and MetS in Korean populations [16,17]. The association is not available for older adults from South Korea. Therefore, the aim of this study was to examine the association between eCRF and MetS in older Korean adults.

## 2. Materials and Methods

### 2.1. Database Source and Study Participants

**Figure 1:**
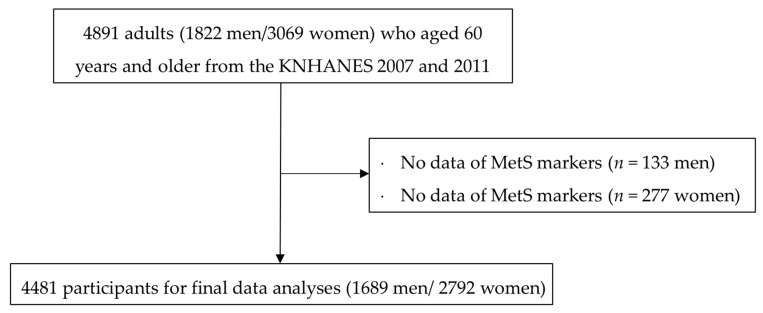
A flowchart for selection of study participants.

The data used in the current study were extracted from the Korea National Health and Nutrition Examination Surveys (KNHANES) IV and V, which were conducted in South Korea from 2008 to 2011 to assess the health-related behaviors, health conditions, and nutritional status of Koreans. A detailed description of the surveys is available elsewhere (http://knhanes.cdc.go.kr/, accessed 3 May 2021). The current study included 1822 men and 3069 women aged 60 years and older (*n* = 4891). Individuals with no data available regarding the components of MetS (133 men and 277 women) were excluded. Consequently, 1689 men and 2792 women (*n* = 4481) were included in the final data analyses (Figure 1). The Institutional Review Board of the Korea Center for Disease Control and Prevention reviewed and approved the KNHANES protocols (Nos. 2008-04EXP-01-C, 2009-01CON-03-2C, 2010-02CON-21-C, 2011-02CON-06C). 

### 2.2. Study Variables

#### 2.2.1. Definition of Metabolic Syndrome (MetS)

MetS was defined according to the following criteria [1] with the acceptance of Korean-specific waist circumference (WC) criteria [18]: (a) abdominal obesity (WC ≥ 90 cm for men and WC ≥ 85 cm for women); (b) elevated TG (TG ≥ 150 mg/dL) or medication use; (c) low HDL-C (HDLC < 40 mg/dL for men and HDLC < 50 mg/dL for women) or medication use; (d) elevated resting blood pressure (SBP ≥ 130 mmHg and/or DBP ≥ 85 mmHg) or use of antihypertensive agents; and (e) elevated fasting glucose (glucose ≥ 100 mg/dL) or use of anti-diabetic medication.

#### 2.2.2. Estimation of Cardiorespiratory Fitness (eCRF)

eCRF was obtained using a previously validated regression equation [11]; eCRF (mL·kg^−1^·min^−1^) = 2.77 × (0 for men, 1 for women) − 0.10 × (age) − 0.17 × (body mass index) − 0.03 × (resting heart rate) + 1.00 × (physical activity score) + 18.07. Physical activity scores were assessed using the Johnson Space Center physical activity rating scale [19]. Individual eCRF values were categorized as low (lowest 25 percentile), middle (middle 50 percentile), and high (highest 25 percentile).

#### 2.2.3. Clinical and Laboratory Measurements

A standardized questionnaire was used to assess health behaviors and socioeconomic status, including smoking, alcohol consumption, physical activity, income, marital status, education, and past medical history. Systolic blood pressure (SBP), diastolic blood pressure (DBP), and resting heart rate (RHR) were also measured using a standardized protocol. WC was measured midway between the highest point of the iliac crest and the bottom of the ribcage. Body mass index (BMI) was calculated as weight (kg) divided by height (m^2^). A detailed description of the clinical measurements is available elsewhere [16]. 

Blood samples were collected from the anterior cubital vein after overnight fasting for the assessments of glucose, total cholesterol (TC), triglycerides (TG), high-density lipoprotein cholesterol (HDLC), insulin, alanine aminotransferase (ALT), aspartate aminotransferase (AST), and vitamin D. The homeostasis model assessment of insulin resistance (HOMA-IR) was calculated; HOMA-IR = fasting insulin (U/L) × fasting glucose (nmol/L)/22.5. A detailed explanation of the laboratory measurements is available elsewhere [20].

### 2.3. Statistics

Prior to statistical analyses, a normality check for all variables was conducted with the Kolmogorov–Smirnov test, and an appropriate transformation was performed if necessary. All data are presented as means and standard deviations for continuous variables and as percentages for categorical variables. Parametric and non-parametric statistics were used to compare continuous and categorical variables, respectively. Logistic regression analyses were used to calculate odds ratios (ORs) and 95% confidence intervals (CIs). Alpha was set at 0.05. All statistical analyses were performed using SPSS-PC statistical software (version 27.0, SPSS, Inc., Chicago, IL, USA).

## 3. Results

Table 1 describes the characteristics of the study participants. Prevalence of MetS was 38.5% (*n* = 1882) in total, 34.5% in men (*n* = 583), and 46.5 (*n* = 1299) in women (*p* < 0.001 for gender). 

Men with MetS were younger, had higher values of BMI (*p* = 0.001), percentage body fat (*p* < 0.001), WC (*p* < 0.001), SBP (*p* < 0.001), and DBP (*p* < 0.001) in conjunction with lower levels of eCRF (*p* < 0.001) and educational background (*p* = 0.003) compared to men without MetS. Men with MetS had higher rates of hypertension and diabetes in conjunction with less favorable blood chemistry profiles compared to men without MetS.

Likewise, women with MetS were older (*p* = 0.002), had higher values of BMI (*p* < 0.001), percentage body fat (*p* < 0.001), WC (*p* < 0.001), RHR (*p* < 0.001), SBP (*p* < 0.001), and DBP (*p* < 0.001) in conjunction with lower levels of eCRF (*p* < 0.001) and educational background (*p* < 0.001) compared to women without MetS. Women with MetS had higher rates of hypertension (*p* < 0.001) and diabetes (*p* < 0.001) in conjunction with less favorable blood chemistry profiles compared to women without MetS. 

In total, individuals with MetS had higher levels of BMI (*p* < 0.001), percentage body fat (*p* < 0.001), WC (*p* < 0.001), SBP (*p* < 0.001), DBP (*p* < 0.001), hypertension (*p* < 0.001), diabetes (*p* < 0.001), and had lower levels of eCRF (*p* < 0.001), educational background (*p* < 0.001), and having a spouse (*p* < 0.001) compared to individuals without MetS. In addition, individuals with MetS had less favorable blood chemistry profiles compared to individuals without MetS. Together, the current findings suggest that regardless of gender, individuals with MetS had higher body fatness and lower cardiorespiratory fitness in conjunction with less favorable blood chemistry profiles compared to individuals without MetS.

Table 2 shows the associations between eCRF and measured parameters. In men, there were decremental linear trends in age (*p* < 0.001), BMI (*p* < 0.001), percentage body fat (*p* < 0.001), WC (*p* < 0.001), SBP (*p* < 0.001), and DBP (*p* = 0.046) across the incremental eCRF quantile (from low to high). There were incremental linear trends in income (*p* = 0.007), marriage (*p* < 0.001), educational background (*p* < 0.001), and decremental linear trends in smoking (*p* < 0.001), alcohol consumption (*p* = 0.039), hypertension (*p* = 0.011), and diabetes (*p* = 0.005) by eCRF levels. There were decremental linear trends in fasting blood glucose (*p* = 0.018), insulin (*p* < 0.001), HOMA-IR (*p* < 0.001), TG (*p* = 0.002), and the prevalence of MetS (*p* < 0.001) in conjunction with incremental linear trends in HDLC (*p* < 0.001) and vitamin D (*p* < 0.001) by eCRF levels. No such linear trends were found in DBP, TC, TC, AST, and ALT by eCRF levels. 

In women, there were significant decremental linear trends in age (*p* < 0.001), BMI (*p* < 0.001), percentage body fat (*p* < 0.001), WC (*p* < 0.001), and SBP (*p* < 0.001) according to incremental eCRF quantile (from low to high). There were incremental linear trends in marriage (*p* < 0.001) and educational background (*p* < 0.001), and decremental linear trends in hypertension (*p* < 0.001) and diabetes (*p* = 0.009) across incremental eCRF levels (from low to high). There were decremental linear trends in fasting blood glucose (*p* < 0.001), insulin (*p* < 0.001), HOMA-IR (*p* < 0.001), TG (*p* < 0.001), and the prevalence of MetS (*p* < 0.001) in conjunction with incremental linear trends in HDLC (*p* < 0.001) and vitamin D (*p* < 0.001) by eCRF levels. No such linear trends were found in DBP, income, smoking, alcohol consumption, TC, AST, and ALT by eCRF levels. 

In total, there were decremental linear trends in age (*p* < 0.001), BMI (*p* < 0.001), percentage a body fat (*p* < 0.001), WC (*p* < 0.001), SBP (*p* < 0.001), and DBP (*p* = 0.046) across the incremental eCRF quantile (from low to high). There were incremental linear trends in marriage (*p* < 0.001), educational background (*p* < 0.001), and decremental linear trends in the prevalence of hypertension (*p* < 0.001) and diabetes (*p* < 0.001) by eCRF levels. There were decremental linear trends in fasting blood glucose (*p* < 0.001), insulin (*p* < 0.001), HOMA-IR (*p* < 0.001), TG (*p* < 0.001), AST (*p* = 0.010), ALT (*p* < 0.001), and the prevalence of MetS (*p* < 0.001) in conjunction with incremental linear trends in HDLC (*p* < 0.001) and vitamin D (*p* < 0.001) by eCRF levels. No such linear trends were found in household income, smoking, alcohol consumption, and TC by eCRF levels. Together, the current findings suggest that regardless of gender, higher eCRF is associated with better body composition and more favorable profile of metabolic risk factors.

Table 3 represents the odds ratios and 95% confidence intervals of abnormalities in the components of MetS by eCRF quantile. In men, ORs for abnormalities in components of MetS, including abdominal obesity (*p* < 0.001), elevated fasting glucose (*p* < 0.001), elevated TG (*p* < 0.001), and low HDLC (*p* < 0.001), decreased linearly by incremental eCRF levels (from low to high).

In women, ORs for abnormalities in components of MetS, including abdominal obesity (*p* < 0.001), elevated fasting glucose (*p* < 0.001), and elevated TG (*p* < 0.001), decreased linearly by eCRF levels (from low to high). No such linear trends in hypertension and low HDLC were found by incremental eCRF levels.

In total, ORs for abnormalities in components of MetS, including abdominal obesity (*p* < 0.001), elevated fasting glucose (*p* < 0.001), elevated TG (*p* < 0.001), and low HDLC (*p* < 0.001), decreased linearly by incremental eCRF levels (from low to high). In general, individuals with higher eCRF were less likely to have abnormalities in several components of MetS, as compared to individuals with lower eCRF. Together, the current findings suggest that higher eCRF is associated with a lower risk for having abnormal components of MetS.

**Figure 2:**
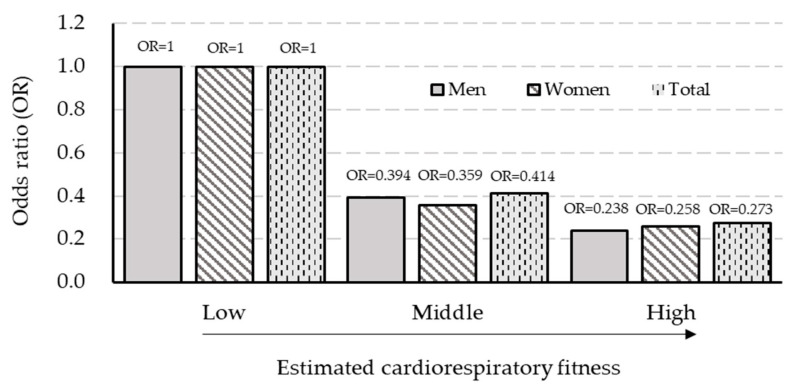
The association between estimated cardiorespiratory fitness and the prevalence of metabolic syndrome. Odds ratio (OR) adjusted for age, sex (total), income, education, marital status, smoking, and alcohol intake.

Figure 2 shows an independent association between eCRF and the prevalence of MetS. Overall, there was a decremental linear trend (F_(2,3980)_ = 116.721, *p* < 0.001) in the prevalence of MetS by incremental eCRF levels (from low to high); regardless of gender, individuals with higher eCRF were less likely to have a MetS diagnosis compared to individuals with lower eCRF.

## 4. Discussion

This population-based study examined whether eCRF is inversely associated with MetS in Korean older adults and showed that low eCRF was significantly associated with less favorable profiles of MetS risk factors. Low eCRF was associated with abnormalities in several components of MetS in both men and women. Regardless of gender, low eCRF was independently associated with a higher risk for having a MetS diagnosis. 

The current findings of this study are consistent with the findings of previous studies involving older adults. In a 2-year follow-up study involving 1226 men and women aged 57–78 years, for example, Hassinen et al. [21] examined the relationship between objectively measured CRF and the development and resolution of MetS and showed that baseline fitness was inversely associated with MetS risk at baseline and higher resolution of MetS over 2 years of follow-up. In a cross-sectional study involving 184 elderly community-dwelling persons aged 60–80 years, Câmara et al. [22] examined the relationships between CRF and muscle strength and MetS and showed that coexistence of low CRF and low muscle strength was an independent predictor of increased MetS risk. Likewise, Arovah et al. [23] examined the relationship between CRF and MetS in a sample of 161 Indonesian middle-aged and older adults and found that low CRF was independently associated with decreased HDL-C, hypertriglyceridemia, and higher MetS prevalence. Taken together, those current and previous findings support the prognostic role of CRF in MetS in geriatric populations.

Even as a well-established risk factor for MetS, CRF is not regularly checked in most healthcare settings because of its impracticality and high cost [24], especially in sedentary elderly persons at risk for mobility disabilities [25]. Alternatively, eCRF can be used as a prognostic tool for risk stratification. For example, in our previous study, we examined the relationship between eCRF and MetS in Korean adults and found that high eCRF was correlated with lower MetS risk [14]. In addition to morbidity, eCRF is associated with premature death from all including specific causes [26,27]. Taken together, those current and previous findings suggest that eCRF has a prognostic value for initial risk stratification in geriatric populations.

The prognostic value of eCRF for MetS diagnosis might reflect the clinical implications of abnormalities in the parameters used for its estimation algorithm, including age, sex, RHR, PA, and BMI. In particular, RHR, PA, and BMI were indicative of healthy lifestyle habits. In a population-based cohort study, Jian et al. [28] examined the association between RHR and MetS and showed that elevated RHR was an independent predictor for MetS diagnosis at baseline and future incidence of MetS in Chinese adults. Likewise, elevated RHR was positively and independently associated with several components of MetS, such as abdominal obesity, hypertension, and hyperglyceridemia, and the presence of MetS in Korean adults [29,30]. In addition to elevated RHR, overweight/obesity and physical inactivity are two well-established risk factors for MetS and related disorders in different populations [31,32].

Several explanations can be given to the protective effect of high eCRF against MetS observed in the current study. First, insulin resistance is a key mechanism underlying MetS pathology. In contrast, high CRF contributes to enhanced insulin sensitivity and/or insulin action in both overweight/obese and normal subjects [33,34]. Second, low-grade inflammation is associated with the etiology of MetS. In contrast, high CRF protects from MetS by suppressing pro-inflammation and enhancing anti-inflammation [35,36]. Lastly, defective mitochondrial biogenesis is another mechanism underlying MetS pathology in skeletal muscle, liver, and pancreas, in conjunction with impaired insulin signaling and glucose metabolism. On the other hand, high CRF protects from MetS by enhancing insulin sensitivity, glucose metabolism, and fatty acid oxidation [37,38].

This study had several limitations. The cross-sectional nature of the current study is a limitation in drawing conclusions regarding causation. The current findings of the study should be confirmed in a cause-and-effect manner by conducting an exercise intervention study. Second, elderly persons are at an especially increased risk of developing MetS due to various physiological mechanisms associated with normal aging, including sarcopenic obesity, lack of physical activity, increased sitting time, and others, which should all be considered in a future study for better delineation of geriatric MetS etiology. Lastly, the gender differences in the relationships between eCRF and specific components of the MetS (i.e., hyperglyceridemia and low HDLC) observed in the current study remain to be further investigated in a future study.

## 5. Conclusions

In conclusion, we examined the association between eCRF and MetS in a representative sample of Korean older adults and found that low eCRF was significantly and independently correlated with abnormalities in components of MetS and its prevalence. The current findings suggest that maintaining and/or obtaining higher CRF via physical activity may be beneficial for older persons who are at an increased risk for MetS.

## Figures and Tables

**Table 1:** Description of study participants by metabolic syndrome status.

	MetS (men)		*p* Value	MetS (Women)		*p* Value	MetS (Total)		*p* Value
	Absence (*n* = 1106)	Presence (*n* = 583)		Absence (*n* = 1493)	Presence (*n* = 1299)		Absence (*n* = 2599)	Presence (*n* = 1882)	
Anthropometrics/hemodynamics									
Age (year)	69.5 ± 6.3	68.5 ± 6.1	0.001	68.8 ± 6.4	69.6 ± 6.1	0.002	69.1 ± 6.4	69.2 ± 6.1	0.522
BMI (kg/m^2^)	22.5 ± 2.7	25.1 ± 2.6	<0.001	23.1 ± 2.9	25.6 ± 3.2	<0.001	22.8 ± 2.8	25.5 ± 3.0	<0.001
Body fat (%)	21.3 ± 5.3	25.3 ± 4.6	<0.001	32.6 ± 5.9	35.9 ± 4.9	<0.001	27.8 ± 8.0	32.6 ± 6.9	<0.001
WC (cm)	81.9 ± 8.7	90.7 ± 7.4	<0.001	79.1 ± 8.3	88.1 ± 8.4	<0.001	80.3 ± 8.5	88.9 ± 8.2	<0.001
RHR (beats/min)	69.8 ± 9.9	70.4 ± 9.7	0.249	70.6 ± 9.1	72.2 ± 9.4	<0.001	57.6 ± 12.3	62.3 ± 17.9	0.004
SBP (mmHg)	126.1 ± 17.4	135.6 ± 15.5	<0.001	125.5 ± 17.1	137.1 ± 16.2	<0.001	125.3 ± 17.3	136.4 ± 16.0	<0.001
DBP (mmHg)	75.7 ± 9.9	80.0 ± 9.7	<0.001	75.2 ± 9.5	79.4 ± 9.7	<0.001	75.4 ± 9.7	79.6 ± 9.7	<0.001
eCRF (METs)	9.3 ± 1.6	8.9 ± 1.5	<0.001	6.4 ± 1.5	5.7 ± 1.5	<0.001	7.6 ± 2.1	6.7 ± 2.1	<0.001
Socioeconomic status									
Income (10,000 won/month)	229 ± 635	213 ± 258	0.572	200 ± 465	177 ± 303	0.128	212.3 ± 544.2	188.1 ± 290.3	0.082
Marital status, *n* (%)			0.330			0.088			<0.001
Married	1024 (92.6)	531 (91.7)		862 (57.7)	696 (53.6)		1886 (72.6)	1227 (65.2)	
Widow/divorced	80 (7.2)	49 (8.4)		625 (41.9)	597 (46.0)		705 (27.1)	646 (34.3)	
Unmarried	2 (0.2)	3 (0.2)		6 (0.4)	6 (0.5)		8 (0.3)	9 (0.5)	
Education, *n* (%)			0.003			<0.001			<0.001
Elementary	510 (46.1)	228 (39.1)		1155 (77.4)	1096 (84.4)		1665 (64.1)	1324 (70.4)	
Middle/high	439 (39.7)	281 (48.2)		293 (19.6)	185 (14.2)		732 (28.2)	466 (24.8)	
College	157 (14.2)	74 (12.7)		45 (3.0)	18 (1.4)		202(7.8)	92 (4.9)	
Health behaviors and conditions									
Smoking, *n* (%)	905 (81.8)	468 (80.3)	0.238	128 (8.6)	115 (8.9)	0.423	1033 (39.7)	583 (31.0)	<0.001
Alcohol, *n* (%)	156 (14.1)	72 (12.3)	0.177	629 (42.1)	581 (44.7)	0.090	785 (30.2)	653 (34.7)	<0.001
Hypertension, *n* (%)	384 (34.7)	329 (56.4)	<0.001	584 (39.1)	795 (61.2)	<0.001	968 (46.3)	1124 (53.7)	<0.001
Diabetes, *n* (%)	136 (12.3)	167 (28.6)	<0.001	123 (8.2)	358 (27.6)	<0.001	277 (10.8)	610 (33.3)	<0.001
Blood chemistry profiles									
FBG (mg/dL)	98.9 ± 24.5	115.5 ± 34.9	<0.001	95.2 ± 18.0	112.2 ± 30.0	<0.001	96.8 ± 21.1	113.6 ± 31.6	<0.001
Insulin (uIU/L)	8.5 ± 4.1	11.8 ± 6.7	<0.001	9.1 ± 4.6	12.6 ± 9.1	<0.001	8.9 ± 4.4	12.3 ± 8.4	<0.001
HOMA-IR	2.1 ± 1.2	3.4 ± 2.5	<0.001	2.2 ± 1.2	3.6 ± 4.1	<0.001	2.1 ± 1.2	3.6 ± 3.7	<0.001
TC (mg/dL)	181.5 ± 34.1	186.3 ± 37.5	0.008	197.2 ± 35.1	202.8 ± 38.0	<0.001	190.5 ± 35.6	197.7 ± 38.6	<0.001
HDLC (mg/dL)	47.0 ± 10.7	37.6 ± 7.9	<0.001	50.4 ± 10.9	42.1 ± 8.3	<0.001	48.9 ± 10.9	40.7 ± 8.5	<0.001
TG (mg/dL)	106.4 ± 51.3	201.9 ± 110.0	<0.001	109.9 ± 52.4	185.0 ± 94.8	<0.001	108.4 ± 51.9	190.2 ± 100.0	<0.001
AST (IU/L)	23.3 ± 7.7	25.2 ± 12.4	<0.001	22.5 ± 8.3	23.3 ± 9.2	0.025	22.9 ± 8.1	23.9 ± 10.3	<0.001
ALT (IU/L)	20.1 ± 10.3	25.8 ± 15.3	<0.001	17.9 ± 11.3	21.2 ± 12.3	<0.001	18.8 ± 11.0	22.6 ± 13.5	<0.001
Vitamin D (ng/mL)	21.9 ± 7.4	20.3 ± 7.3	<0.001	19.2 ± 7.5	18.6 ± 7.3	0.026	20.4 ± 7.6	19.1 ± 7.4	<0.001

MetS: metabolic syndrome; BMI: body mass index; WC: waist circumference; RHR: resting heart rate; SBP: systolic blood pressure; DBP: diastolic blood pressure; eCRF: estimated cardiorespiratory fitness; FBG: fasting blood glucose; HOMA-IR: homeostatic model assessment for insulin resistance; TC: total cholesterol; HDLC: high density lipoprotein-cholesterol; TG: triglycerides; AST: aspartate aminotransferase; ALT: alanine aminotransferase.

**Table 2:** Descriptive statistics of measured parameters by estimated cardiorespiratory fitness.

Variable	Levels of eCRF (Men)			*p* for Trends	Levels of eCRF (Women)			*p* for Trends	Levels of eCRF (Total)			*p* for Trends
	Low	Middle	High		Low	Middle	High		Low	Middle	High	
Age (years)	75.3 ± 5.8	68.1 ± 5.5	66.1 ± 4.9	<0.001	75.8 ± 5.8	68.0 ± 5.6	66.1 ± 4.9	<0.001	72.1 ± 7.6	69.1 ± 6.1	68.4 ± 5.9	<0.001
BMI (kg/m^2^)	24.4 ± 3.0	23.1 ± 3.0	22.7 ± 2.7	<0.001	25.6 ± 3.8	23.8 ± 3.1	23.6 ± 2.9	<0.001	26.3 ± 3.2	23.5 ± 3.0	22.7 ± 2.7	<0.001
BF (%)	25.2 ± 5.2	22.4 ± 5.3	20.9 ± 5.2	<0.001	36.1 ± 5.7	33.9 ± 5.5	32.7 ± 5.9	<0.001	33.2 ± 7.2	29.7 ± 7.7	27.8 ± 8.1	<0.001
WC (cm)	88.0 ± 8.9	84.4 ± 9.6	82.3 ± 8.4	<0.001	87.2 ± 10.0	82.3 ± 8.7	80.9 ± 8.8	<0.001	90.1 ± 9.0	82.9 ± 8.9	80.1 ± 8.3	<0.001
SBP	131.6 ± 16.9	127.5 ± 17.1	127.5 ± 17.5	<0.001	134.4 ± 17.5	130.4 ± 17.7	128.3 ± 17.7	<0.001	132.3 ± 17.2	129.7 ± 17.7	128.6 ± 17.5	<0.001
DBP	76.2 ± 9.9	77.5 ± 10.0	77.7 ± 10.0	0.058	76.8 ± 10.2	77.1 ± 9.8	77.0 ± 10.0	0.710	77.8 ± 9.9	77.0 ± 10.6	76.7 ± 10.1	0.046
Income (10,000 won/month)	154 ± 439	228 ± 527	285 ± 776	0.007	228 ± 758	224 ± 1413	187 ± 256	0.713	196.3 ± 452.9	230.5 ± 1123.1	205.2 ± 554.7	0.576
Marital status, *n* (%)				<0.001				<0.001				<0.001
Married	342 (86.4)	727 (93.0)	384 (94.3)		239 (33.7)	832 (59.0)	464 (65.7)		515 (58.5)	1832 (69.2)	641 (72.8)	
Widow/divorced	54 (13.6)	61 (6.5)	22 (5.4)		466 (65.7)	573 (40.7)	240 (34.0)		363 (41.2)	808 (30.5)	235 (26.7)	
Unmarried	0 (0)	4 (0.5)	1 (0.2)		4 (0.6)	4 (0.3)	2 (0.3)		2 (0.2)	9 (0.3)	4 (0.5)	
Education, *n* (%)				<0.001				<0.001				<0.001
Elementary	218 (55.1)	340 (43.5)	148 (36.4)		637 (89.8)	1125 (79.8)	546 (77.3)		669 (76.0)	1756 (66.3)	589 (56.9)	
Middle/high	133 (33.6)	350 (44.8)	186 (45.7)		69 (9.7)	248 (17.6)	138 (19.5)		712 (19.5)	724 (27.3)	228 (25.9)	
College	45 (11.4)	92 (11.8)	73 (17.9)		3 (0.4)	36 (2.6)	22 (3.1)		39 (4.4)	169 (6.4)	63 (7.2)	
Smoking, *n* (%)	88 (12.4)	115 (8.2)	40 (5.7)	<0.001	320 (80.8)	641 (82.0)	324 (79.6)	0.607	296 (33.6)	948 (35.8)	284 (32.3)	0.128
Alcohol, *n* (%)	343 (48.4)	809 (42.6)	396 (43.9)	0.039	66 (16.7)	103 (13.2)	51 (12.5)	0.172	318 (36.1)	860 (32.5)	295 (33.5)	0.135
Hypertension, *n* (%)	448 (63.2)	677 (48.0)	290 (41.1)	0.011	202 (51.0)	321 (41.0)	143 (35.1)	<0.001	522 (59.3)	1207 (45.6)	352 (40.0)	<0.001
Diabetes, *n* (%)	154 (21.7)	236 (16.7)	100 (14.2)	0.005	87 (22.0)	153 (19.6)	53 (13.0)	0.009	214 (29.4)	470 (19.5)	127 (15.3)	<0.001
FBG (mg/dL)	107.9 ± 33.4	106.5 ± 33.2	101.9 ± 23.2	0.018	108.5 ± 30.3	102.2 ± 25.5	101.1 ± 23.4	<0.001	109.9 ± 32.2	103.7 ± 28.2	100.2 ± 21.6	<0.001
Insulin (uIU/L)	11.8 ± 7.9	9.2 ± 4.1	8.5 ± 3.5	<0.001	12.8 ± 12.4	10.4 ± 5.4	9.7 ± 4.4	<0.001	12.6 ± 7.4	10.2 ± 7.2	8.8 ± 3.6	<0.001
HOMA-IR	3.2 ± 2.8	2.5 ± 1.6	2.2 ± 1.1	<0.001	3.6 ± 5.6	2.7 ± 2.0	2.5 ± 1.5	<0.001	3.5 ± 2.7	2.7 ± 3.1	2.2 ± 1.2	<0.001
TC (mg/dL)	183.5 ± 35.0	184.8 ± 35.3	183.6 ± 36.7	0.808	202.1 ± 37.5	199.2 ± 36.1	200.0 ± 36.3	0.300	195.5 ± 38.4	194.5 ± 36.7	192.7 ± 36.5	0.294
HDLC (mg/dL)	41.7 ± 10.4	43.2 ± 10.6	46.1 ± 11.0	<0.001	45.2 ± 9.7	46.6 ± 10.7	47.6 ± 10.8	<0.001	43.5 ± 10.1	45.6 ± 10.8	46.8 ± 10.8	<0.001
TG (mg/dL)	145.5 ± 86.6	145.1 ± 92.9	127.1 ± 83.9	0.002	153.0 ± 74.2	146.7 ± 87.3	135.3 ± 79.1	<0.001	161.9 ± 97.9	142.9 ± 81.0	128.4 ± 81.5	<0.001
AST (IU/L)	23.6 ± 8.2	24.2 ± 11.2	24.0 ± 7.9	0.648	23.0 ± 8.2	22.7 ± 7.7	23.4 ± 10.9	0.202	24.1 ± 9.3	23.0 ± 8.7	23.5 ± 10.2	0.010
ALT (IU/L)	22.4 ± 13.3	22.7 ± 13.7	21.0 ± 9.3	0.092	19.3 ± 11.3	19.2 ± 10.3	20.1 ± 14.9	0.243	23.0 ± 13.9	19.9 ± 11.1	19.7 ± 13.5	<0.001
Vitamin D (ng/mL)	20.0 ± 7.1	21.4 ± 7.6	22.8 ± 7.5	<0.001	17.8 ± 7.3	19.0 ± 7.4	20.0 ± 7.8	<0.001	18.9 ± 7.4	19.7 ± 7.4	21.2 ± 8.0	<0.001
MetS, *n* (%)	376 (64.3)	579 (44.2)	252 (37.0)	<0.001	376 (64.3)	579 (44.2)	252 (37.0	<0.001	464 (61.9)	997 (40.5)	264 (31.3)	<0.001

BMI: body mass index; WC: waist circumference; SBP: systolic blood pressure; DBP: diastolic blood pressure; FBG: fasting blood glucose; HOMA-IR: homeostatic model assessment for insulin resistance; TC: total cholesterol; HDLC: high density lipoprotein-cholesterol; TG: triglycerides; AST: aspartate aminotransferase; ALT: alanine aminotransferase; MetS: metabolic syndrome.

**Table 3:** Odds ratios (ORs) and 95% confidence intervals (CIs) for abnormal components of metabolic syndrome by estimated cardiorespiratory fitness.

Components	Levels of eCRF (Men)			*p*	Levels of eCRF (Women)			*p*	Levels of eCRF (Total)			*p*
	Low	Middle	High		Low	Middle	High		Low	Middle	High	
Abdominal obesity	1	0.261 (0.191–0.358)	0.131 (0.089–0.197)	<0.001	1	0.185 (0.146–0.235)	0.138 (0.105–0.182)	<0.001	1	0.277 (0.191–0.270)	0.123 (0.098–0.153)	<0.001
Hypertension	1	0.825 (0.625–1.088)	0.938 (0.677–1.302)	0.301	1	0.862 (0.696–1.069)	0.744 (0.581– 0.953)	0.060	1	0.938 (0.800–1.099)	0.896 (0.738–1.087)	0.055
Hyperglycemia	1	0.661 (0.493–0.887)	0.474 (0.335– 0.670)	<0.001	1	0.460 (0.364–0.580)	0.417 (0.319– 0.544)	<0.001	1	0.569 (0.481–0.674)	0.469 (0.382–0.576)	<0.001
Hypertriglyceridemia	1	0.630 (0.462–0.858)	0.347 (0.239–0.504)	<0.001	1	0.708 (0.562–0.891)	0.502 (0.384–0.657)	<0.001	1	0.653 (0.551–0.774)	0.406 (0.327–0.503)	<0.001
Low HDLC	1	0.591 (0.441–0.792)	0.411 (0.291–0.582)	<0.001	1	0.970 (0.761–1.238)	0.901 (0.685–1.187)	0.696	1	0.738 (0.617–0.882)	0.677 (0.547–0.837)	<0.001

HDLC: high density lipoprotein-cholesterol. ORs adjusted for age, sex, income, education, marital status, smoking, and alcohol intake.

## Data Availability

The datasets used and/or analysed during the current study are available from the corresponding author on request.
